# Wheat stripe rust resistance locus *YR63* is a hot spot for evolution of defence genes – a pangenome discovery

**DOI:** 10.1186/s12870-023-04576-2

**Published:** 2023-11-27

**Authors:** Amy Mackenzie, Michael Norman, Mesfin Gessese, Chunhong Chen, Chris Sørensen, Mogens Hovmøller, Lina Ma, Kerrie Forrest, Lee Hickey, Harbans Bariana, Urmil Bansal, Sambasivam Periyannan

**Affiliations:** 1 Commonwealth Scientific and Industrial Research Organization Agriculture and Food, Canberra, Australian Capital Territory 2601 Australia; 2 Centre for Crop Sciences, Queensland Alliance for Agriculture and Food Innovation, The University of Queensland, St Lucia, Brisbane, Queensland 4072 Australia; 3 School of Life and Environmental Sciences, Faculty of Science, The University of Sydney Plant Breeding Institute, 107 Cobbitty Road, Cobbitty, New South Wales 2570 Australia; 4 Present address:, Wolaita sodo University, Sodo, Ethiopia; 5 Department of Agroecology, Aarhus University, Forsøgsvej 1, 4200 Slagelse, Denmark; 6 Agriculture Victoria, Department of Energy, Environment and Climate Action, AgriBio, Centre for AgriBioscience, 5 Ring Rd, Bundoora, Victoria 3083 Australia; 7 School of Science, Western Sydney University, Bourke Road, Richmond, New South Wales 2753 Australia; 8 School of Agriculture and Environmental Science & Centre for Crop Health, University of Southern Queensland, Toowoomba, Queensland 4350 Australia

**Keywords:** Wheat landrace, Stripe rust resistance, Markers, Validation

## Abstract

**Background:**

Stripe rust, caused by *Puccinia striiformis* f. sp. *tritici* (*Pst*), poses a threat to global wheat production. Deployment of widely effective resistance genes underpins management of this ongoing threat. This study focused on the mapping of stripe rust resistance gene *YR63* from a Portuguese hexaploid wheat landrace AUS27955 of the Watkins Collection.

**Results:**

*YR63* exhibits resistance to a broad spectrum of *Pst* races from Australia, Africa, Asia, Europe, Middle East and South America. It was mapped to the short arm of chromosome 7B, between two single nucleotide polymorphic (SNP) markers *sunCS*_*YR63* and *sunCS_67*, positioned at 0.8 and 3.7 Mb, respectively, in the Chinese Spring genome assembly v2.1. We characterised *YR63* locus using an integrated approach engaging targeted genotyping-by-sequencing (tGBS), mutagenesis, resistance gene enrichment and sequencing (MutRenSeq), RNA sequencing (RNASeq) and comparative genomic analysis with tetraploid (Zavitan and Svevo) and hexaploid (Chinese Spring) wheat genome references and 10+ hexaploid wheat genomes. *YR63* is positioned at a hot spot enriched with multiple nucleotide-binding and leucine rich repeat (NLR) and kinase domain encoding genes, known widely for defence against pests and diseases in plants and animals. Detection of *YR63* within these gene clusters is not possible through short-read sequencing due to high homology between members. However, using the sequence of a *NLR* member we were successful in detecting a closely linked SNP marker for *YR63* and validated on a panel of Australian bread wheat, durum and triticale cultivars.

**Conclusions:**

This study highlights *YR63* as a valuable source for resistance against *Pst* in Australia and elsewhere. The closely linked SNP marker will facilitate rapid introgression of *YR63* into elite cultivars through marker-assisted selection. The bottleneck of this study reinforces the necessity for a long-read sequencing such as PacBio or Oxford Nanopore based techniques for accurate detection of the underlying resistance gene when it is part of a large gene cluster.

**Supplementary Information:**

The online version contains supplementary material available at 10.1186/s12870-023-04576-2.

## Background

Rust diseases are one of the major threats to global wheat production. The emergence and spread of highly virulent strains of *Puccinia striiformis* f. sp. *tritici* (*Pst*) that causes wheat stripe rust has had a significant contribution towards its impact [1]. In Australia, *Pst* pathotypes detected so far belongs to four lineages, namely 104 E137 A- (belongs to global *Pst* lineage classification *Pst*S0), 134 E16 A+ (*Pst*S1), 198 E16 A+ J+ T+ 17+ (*Pst*S13) and 239 E237 A- 17+ 33+ (*Pst*S10). Interestingly, these pathotypes, each with its own unique virulence pattern, entered Australia through four independent incursion events [2]. These events made breeding for stripe rust resistance a highly challenging task as new resistance gene combinations are required to be effective against these distinct pathotypes. While genes belonging to adult plant resistance (*APR*) class are broadly effective, their expression only at the adult plant stages does not provide protection at the seedling and early juvenile plant growth stages. Hence, deployment of *APR* genes along with widely effective all-stage resistance (*ASR*) genes remains essential for protecting wheat crops against rust diseases [3].

Most of the cloned *ASR* genes encode nucleotide-binding-leucine-rich-repeat (NLR) proteins [4]. NLRs typically have three domains: an N-terminal coiled coil (CC) or Toll/Interleukin-1 receptor (TIR), a C-terminal leucine-rich repeat (LRR) and a central nucleotide-binding (NB) domain [5]. The recently developed mutagenesis, resistance gene enrichment and sequencing (MutRenSeq) approach is a powerful tool for *ASR* gene discovery and it relies on the assumption that most *ASR* genes encode NLR proteins. While there is a chance that the *NLR* bait library is not extensive enough to capture all *NLRs*, this technique unfortunately will not detect any non-*NLR* coding genes [6]. More recent research has revealed two more types of *ASR* genes; tandem kinases, and transmembrane proteins with ankyrin domains [4]. The *ASR* gene *Yr15* and *Sr60,* encode a tandem kinase (ie. a protein containing two kinase domains), which confers strong and partial resistance to wheat stripe rust and stem rust, respectively [7, 8]. Similarly, the leaf rust *ASR* gene *Lr14a* was found to encode an ankyrin-transmembrane protein [9]. Hence it is important not to limit the search for new *ASR* genes to *NLR* class alone.

In such cases map-based approach paired with comparative genomics will be more appropriate. While the annotated International Wheat Genome Sequencing Consortium (IWGSC) RefSeq v2.1 genome of Chinse Spring wheat is a valuable resource, there is a large degree of unexplored diversity in other wheat varieties [10]. Over the last few years, several hexaploid wheat cultivars have been sequenced and annotated by the “10+ Wheat Genomes Project” to develop a wheat pangenome [11]. In addition, reference genomes are also available for tetraploid wheat cultivars Svevo [12] and Zavitan (WEWseq v1.0) [13], and closely related diploid grasses.

The available common wheat landrace collections including the “Watkins Collection” representing over 32 wheat-producing nations have been a valuable resource to discover widely effective stripe rust resistance genes [14–16]. One of the stripe rust resistance genes identified in a Portuguese hexaploid wheat landrace AUS27955 [Australian gene bank (AGG) No: AGG27955WHEA1] from the Watkins Collection was located on the short arm of chromosome arm 7B and it was named *YR63* (Bansal and Bariana, unpublished results). The gene exhibited resistance against all known Australian *Pst* pathotypes, except 239 E237 A- 17+ 33+ within the *Pst*S10 lineage.

Here, we screened *YR63* against globally important *Pst* isolates at the Global Rust Reference Center, Denmark to understand its broad-spectrum nature. We employed tGBS, MutRenSeq, RNA sequencing (RNASeq) and comparative genomic analysis to fine map and identify molecular markers closely linked with *YR63*.

## Results

### Effectiveness of *YR63* against multiple *Pst* pathotypes

**Fig. 1:**
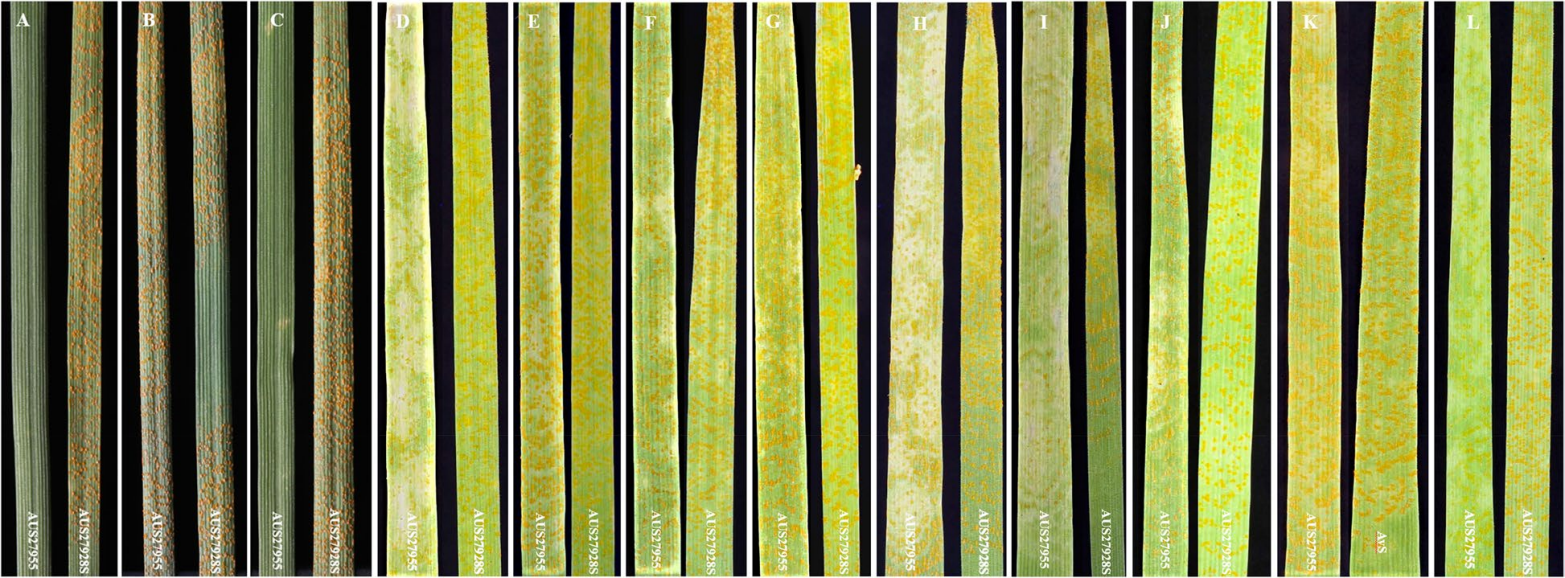
Infection of AUS27955 and AUS27928S against *Pst* pathotypes. Australian: *Pst*S1 (**A**), *Pst*S10 (**B**), *Pst*S13 (**C**). Global: *Pst*S2 (**D**)*, Pst*S7 (**E**)*, Pst*S8 (**F**)*, Pst*S9 (**G**)*, Pst*S10 (**H**)*, Pst*S11 (**I**), *Pst*S13 (**J**), *Pst*S14 (AUS27928S unavailable and replaced with Avocet ‘S’ (AvS)) (**K**) and *Pst*S17 (**L**)

Against Australian *Pst* pathotypes, the *YR63* donor accession AUS27955 produced infection type (IT) ‘0;’ against *Pst*S1 (Figure 1A) and *Pst*S13 (Figure 1B), while a susceptible response (IT ‘3+’) similar to the susceptible parent AUS27928S was observed against *Pst*S10 (Figure 1C, Table 1). Among the AUS27955 x AUS27928S-derived recombinant inbred line (RIL) population, 93 lines produced IT ‘0;’ and 102 lines produced IT ‘3+’ against *Pst*S1 pathotype, following an expected single-gene segregation ratio of 1:1 (χ^2^= 0.42, d.f. = 1, *p*-value = 0.5).

**Table 1:** Resistance response of AUS27955 and AUS27928S against Australian and International *Pst* pathotypes

**Region**	***Pst* Genetic Group**	**AUS27955**	**AUS27928S**
Australia	*Pst*S1	;	3+
	*Pst*S10	3+	3+
	*Pst*S13	;	3+
West Asia, North & East Africa	*Pst*S2	2C	3
Europe	*Pst*S7	3+C	3+
Europe	*Pst*S8	2+C	3C
Asia	*Pst*S9	3C	3+
Europe	*Pst*S10	2+C	3+
Asia, Africa	*Pst*S11	2C	3C
South America	*Pst*S13	2+C	3
Africa	*Pst*S14	3+C	3+
Egypt & Turkey	*Pst*S17	2C	3

Tests against 10 global *Pst* pathotypes representing different genetic groups and geographical regions such as Africa, Asia, Middle East and South America, AUS27955 exhibited IT 2C (against *Pst*S2 & *Pst*S11 and *Pst*S17), 3C (*Pst*S9) and 3+C (*Pst*S7 and *Pst*S14) (Figure 1, Table 1).

### Targeted genotyping-by-sequencing (tGBS) analysis positions *YR63* within 0.6 to 7.4 Mb interval of chromosome (Chr) 7B

In the tGBS analysis, a total of 4,442 markers, across the Chr 7 groups of A, B and D genomes were found polymorphic between AUS27955 and AUS27928S. Among them, 11 tGBS markers from the short arm of Chr 7B showed close association with *YR63* and were targeted for SNP based KASP marker analysis. While the tGBS analysis predicted abundant scaffolds, there were only 4 KASP markers namely, *sunKASP_401* (from scaffolds 62788), *sunKASP_406* (scaffold 13660), *sunKASP_409* and *sunKASP_407* (scaffold 96545) showed clear polymorphism between the resistant and susceptible parents and were used for mapping *YR63* on the RIL population (Table 2). Markers s*unKASP_401* and *sunKASP_406* positioned at 0.6 and 7.4 Mb interval of Chinese Spring genome assembly v2.1*,* flanked *YR63* distally and proximally at genetic distances of 4.2 and 16.1 cM, respectively.

**Table 2:** KASP markers used to map *YR63* on chromosome arm 7BS

Marker	Position^a^	Allele 1 Specific Forward Primer	Allele 2 Specific Forward Primer	Common Reverse Primer
*sunKASP_401*	262,576	ATGTTGTGTAGAAATTAGAGAATATGGAGT	GTTGTGTAGAAATTAGAGAATATGGAGC	CACGTGTTCAGCAAAAGGAG
*sunCS_YR63*	904,156	CTGAATCACATCTATTAACCTCCAAATC	CTGAATCACATCTATTAACCTCCAAATG	AAGTTTGTGACTGCCCCAAGAT
*sunCS_67*	4,028,196	GCACCGTTGGTACTATTTAGCAT	GCACCGTTGGTACTATTTAGCAC	CCCCAAGCTTGCTACAGTGTC
*sunCS_36*	5,843,626	AGCTGTAAATAATTGCCTCACCT	AGCTGTAAATAATTGCCTCACCC	GCTACGCGGAAATTTGACCA
*sunKASP_406*	7,984,146	TGCCATCTAGTTGAGTAACCTCTG	AATGCCATCTAGTTGAGTAACCTCTA	CACAAAAACCCCTTCACACC
*sunKASP_409*	13,227,705	CAATGCATTTTCTTCTTCTCCG	CAATGCATTTTCTTCTTCTCCC	CTTCACCACCGCATTCCTA
*sunKASP_407*	13,398,475	AATTGCCCAAGAGGGTCTAA	AATTGCCCAAGAGGGTCTAG	GCGTTTGGGTATCATTCCAC

^a^Based on Chinese Spring (v2.1). Allele 1 primer synthesised with FAM: GAA GGT GAC CAA GTT CAT GCT; Allele 2 primer synthesised with HEX: GAA GGT CGG AGT CAA CGG ATT

### Marker enrichment via RNAseq

**Fig. 2:**
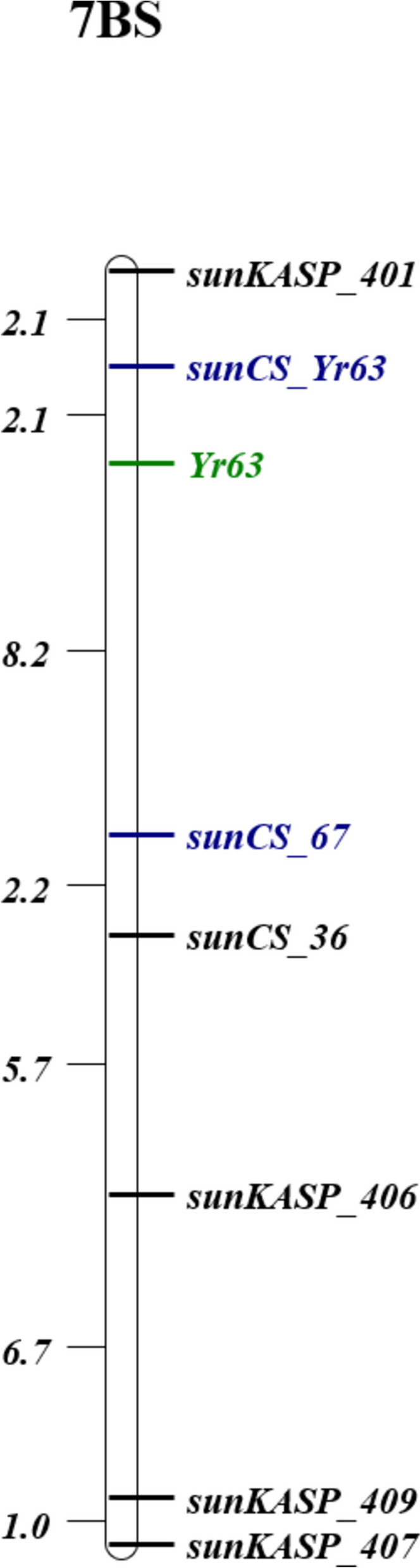
Genetic linkage map of *YR63* locus in AUS27955 x AUS27928S F_6_ RIL population. Distances are shown in cM. n = 195

There were 57 SNPs identified between AUS27955 and AUS27928S from the RNAseq reads related to genes present in the 0.9 - 7.8 Mb interval of the Chinese Spring Chr 7B (IWGSC RefSeq v2.1). A total of 20 SNPs selected at random positions were converted into KASP markers. Only two markers, *sunCS_67* and *sunCS*_*36*, at ~4 and ~5.8 Mb, respectively were found polymorphic and mapped proximal to *YR63*. Sixteen recombinants between the closest marker, *sunCS_67* and *YR63* were detected (Figure 2).

### MutRenSeq reveals a *NLR* gene as a possible candidate for *YR63*

**Fig. 3:**
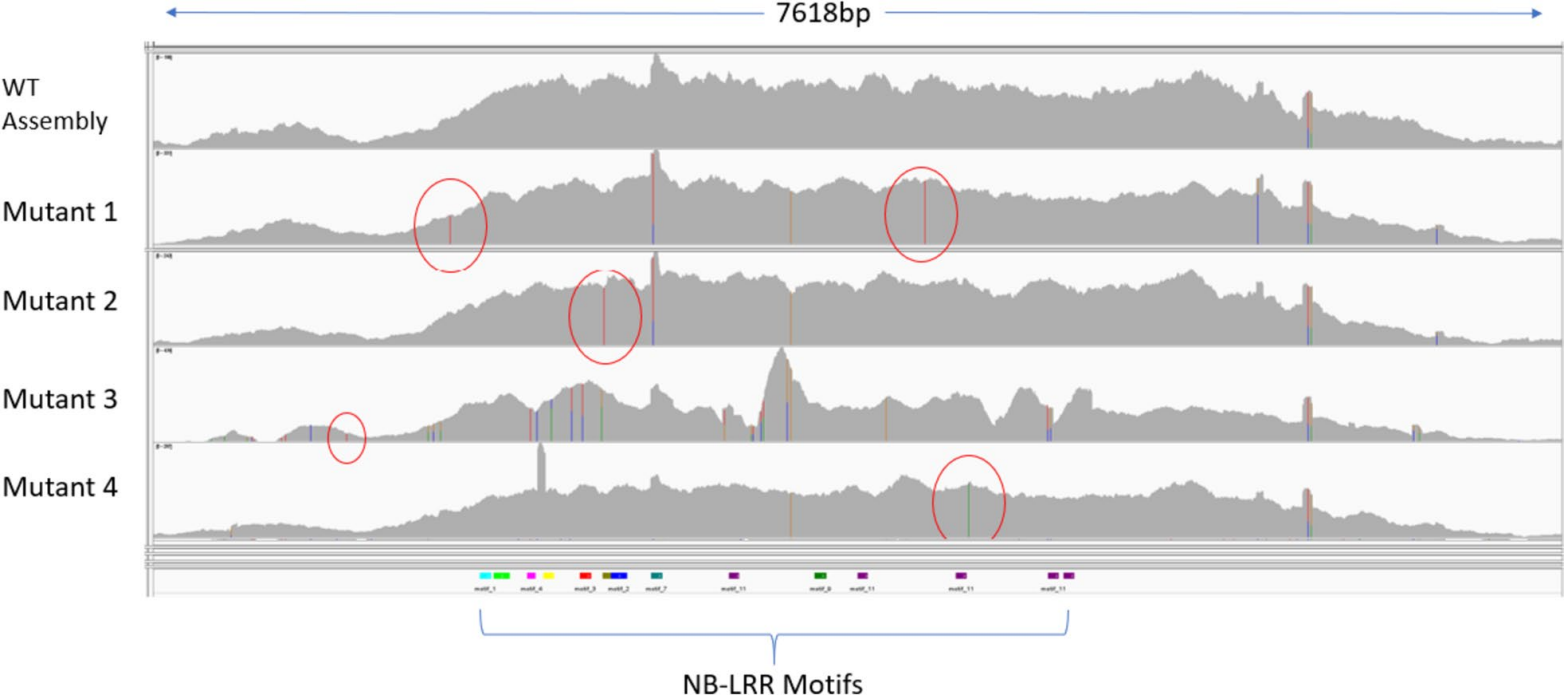
DNA sequence linked with *YR63* resistance identified through MutRenSeq, for four mutants and wild-type (WT) AUS27955, visualised in Integrated Genomics Viewer (IGV) 2.8.7. Unique SNPs are circled in red

Six loss-of-function mutants were identified for *YR63* through Ethyl methanesulphonate (EMS) based mutagenesis. Subsequently in the MutRenSeq, the raw Illumina sequencing reads of the four mutants and the resistant accession AUS27955 had a quality score of Q30 over 90% for all base calls. Quality assessment via FastQC showed one over-represented sequence in the raw data from AUS27955. This sequence was added to the adapter sequences for trimming. Over 93% of the reads survived the trimming step and the trimmed reads were used for the de-novo assembly. The MutRenSeq pipeline revealed one *NLR* contig (Figure 3) that was mutated in three of the four mutants and was related to *TraesCS7B03G0004700*. It mapped near the telomere on Chr arm 7BS (Chr7B:900491-909163) of the IWGSC RefSeq v2.1 Chinese Spring reference [17]. Three of the four mutants contain SNPs within the *NLR* coding sequences (CDS), while mutant 3’s unique SNP is located 20 bp upstream of the start codon in the 5’ untranslated region (UTR). Analysis of the remaining mutants in Geneious Prime showed that all SNPs within the CDS altered the amino acid (‘aa’) sequence. In the case of mutant 1, a premature stop codon was introduced to result in a nonsense mutation within the LRR domain at the ‘aa’ position 1029. In mutant 2, a C>T SNP caused a serine>phenylalanine missense mutation in the NB-ARC domain at ‘aa’ position 451, and in mutant 4, a G>A SNP caused a glycine>arginine missense mutation in the LRR domain at ‘aa’ position 1107. There was a high degree of polymorphisms in mutant 3 compared to the wild-type and other mutants, which suggested several homoeologs of similar genes had collapsed into a single contig during assembly.

### KASP marker from the predicted *NLR* gene doesn’t co-segregate but linked closely with *YR63*

KASP markers were designed for *YR63_NLRC* based on SNPs between the resistant parent (AUS27955) and the IWGSC RefSeq v2.1 reference sequence. Of the markers designed from 8 different SNPs, *sunCS_YR63* located at CDS position 2871 and at 0.9 Mb on Chr 7B of Chinese Spring reference IWGSC RefSeq v2.1 worked well to distinguish between the resistant and susceptible alleles and a heterozygous control (Table 2). Five recombinants were detected between *sunCS_YR63* and *YR63* among the RIL population.

### *YR63* homologous region in pan-genome is enriched with multiple *NLR* and *kinase* genes

As the marker generated from the MutRenSeq did not yield a co-segregating marker, we decided to investigate candidate genes from sources outside the Chinese Spring v2.1. To generate a list of candidate genes, the closest flanking markers *sunCS_YR63* and *sunCS_67* and the genes present in the *YR63* locus from Chinese Spring v2.1*,* were used to BLAST against additional hexaploid wheat and durum reference genomes (Table 3). Unexpectedly, the flanking markers were mapped to Chr 5B instead of Chr 7B in Arina*LrFor* and SY Mattis references and no conclusive region was determined, thus these genomes were removed from the analysis. The size of the *YR63* locus ranged between 1.19 to 3.58 Mb across the pangenome and there were 31 to 141 genes within this interval.

**Table 3:** Pangenome summary for the *YR63* locus

	**Position in Chromosome 7B (bp)**				
**Genome**	***sunCS_YR63***	***sunCS_67***	**Interval (Mb)**	**Orientation**	**No. of Genes**
CDC Landmark	2 601 954	3 792 834	1.19	forward	31
LongReach Lancer	2 383 200	3 628 303	1.25	forward	34
Jagger	2 457 124	3 724 247	1.27	forward	35
Chinese Spring v1.0	886 013	3 700 024	2.81	forward	105
Svevo	888 787	3 735 754	2.85	forward	141
PI190962 (spelt wheat)	1 019 123	3 892 955	2.87	forward	40
CDC Stanley	8 327 844	5 407 847	2.92	reverse	53
Mace	1 161 441	4 087 863	2.93	forward	57
Julius	1 096 280	4 114 505	3.02	forward	52
Chinese Spring v2.1	901 880	4 028 196	3.13	forward	106
Norin	8 596 118	5 458 876	3.14	reverse	45
Zavitan	1 665 643	5 247 144	3.58	forward	44

Twenty-two genes were predicted to encode putative disease resistance proteins where 16 encoded NLRs and the remaining 6 genes encoded a kinase protein with three being annotated as LRR-receptor like protein kinases (Table 4). In the MutRenSeq analysis of the 16 *NLR* candidates, none of the genes showed polymorphism in all four loss-of-function mutants.

**Table 4:** Summary of *NLR* and *kinase* genes detected in the *YR63* pangenome loci

**Gene structure**	**Gene ID**	**Cultivar**	**Length (bp)**	**Encoded protein**	**Upregulated**
*NLR*	*TraesCS7B03G0004800LC*	Chinese Spring, Svevo	630	NB-ARC domain protein	
	*TRITD7Bv1G000550*	Svevo	644	NBS-LRR disease resistance protein-like protein G	
	*TRITD7Bv1G000560*	Svevo	809	NB-ARC domain protein	
	*TraesCS7B03G0007100LC*	Chinese Spring	732	NB-ARC domain protein	
	*TraesMAC7B01G002800*	Mace	1098	NB-ARC domain protein	
	*TraesCS7B03G0005200LC*	Chinese Spring, Svevo	2319	Putative disease-resistance protein	
	*TraesCS7B03G0005300*	Chinese Spring, Norin61, Spelta, Stanley	2469	Putative disease resistance protein	+
	*TraesMAC7B01G002900*	Mace	2862	NB-ARC domain protein	
	*TraesMAC7B01G004100*	Mace	3045	Putative disease-resistance protein	
	*TraesJUL7B01G003200*	Julius	3681	NB-ARC domain protein	
	*TRITD7Bv1G000660*	Svevo	4071	Disease resistance protein (TIR-NBS-LRR)	+
	*TraesJUL7B01G006900*	Julius, Mace, Stanley	4260	Disease resistance protein RGA2-like	
	*TraesCS7B03G0013500*	Chinese Spring	4260	NBS-LRR	
	*TraesCS7B03G0006500*	Chinese Spring, Svevo	5022	NB-ARC LRR protein	+
	*TRIDC7BG000370*	Jagger, Landmark, Lancer, Zavitan	12433	Putative disease resistance protein RGA3-like	+
	*TraesCS7B03G0006700*	Chinese Spring, Mace, Norin61, Stanley, Svevo	14204	NB-ARC LRR protein	
*Kinase*	*TraesCS7B03G0005000LC*	Chinese Spring, Svevo	597	LRR receptor-like protein kinase	
	*TRITD7Bv1G000640*	Svevo	1119	LRR receptor-like protein kinase	
	*TraesCS7B03G0005400*	Chinese Spring, Norin61, Stanley	2437	Kinase protein	
	*TraesCS7B03G0012700*	Chinese Spring, Julius, Mace, Norin61, Spelta, Stanley, Svevo, Zavitan	4546	ATP-dependent 6-phosphofructokinase	+
	*TraesCS7B03G0006400*	Chinese Spring, Julius, Mace, Norin61, Spelta, Stanley, Svevo, Zavitan	6334	Kinase protein	+
	*TRITD7Bv1G000690*	Svevo	6910	LRR receptor-like protein kinase	

+ indicates upregulated

**Fig. 4:**
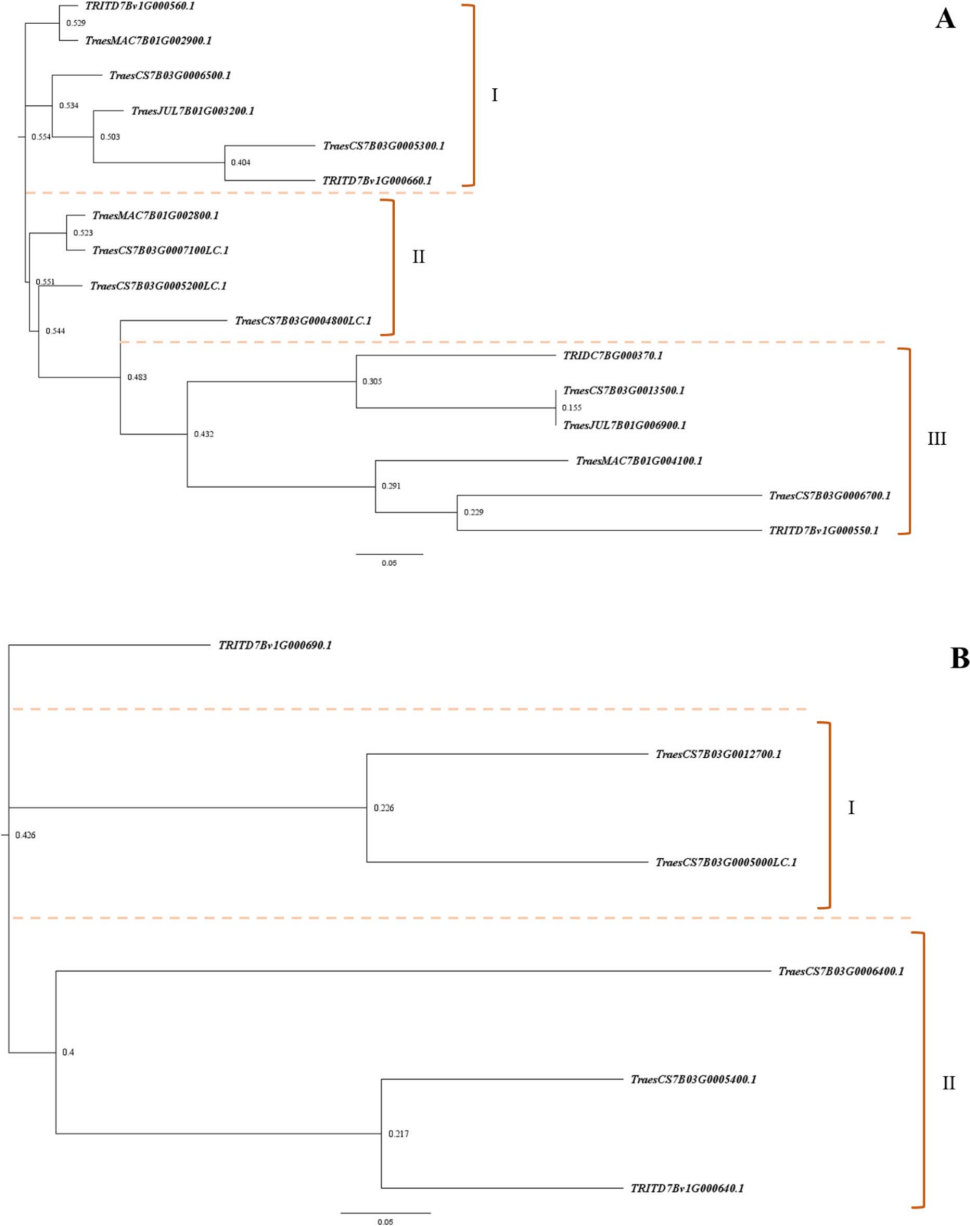
Phylogenetic trees of orthologous and paralogous candidate *NLR* (**A**) and *kinase* (**B**) genes from the *YR63* locus

To determine the genetic relationship within the members of *NLR* and *kinase* genes at the *YR63* locus, we obtained the gene sequences and aligned them using the Clustal-Omega multiple sequence alignment tool. This resulted in the formation of three distinct clusters for the *NLR* genes, of which group I held three of the four upregulated *NLR* genes (Figure 4A). The *kinase* genes formed two clusters, of which each group held a single upregulated *kinase* gene (Figure 4B).

### Marker validation on Australian hexaploid and tetraploid wheat and triticale varieties

The closely linked KASP marker, *sunCS_YR63* of *YR63* was screened against 123, 15 and 14 cultivars of hexaploid and tetraploid wheat and triticale, respectively. Interestingly, the marker was able to distinguish AUS27955 from all the tested varieties indicating its suitability for marker-assisted selection of *YR63* carrying lines (Table 5; Supplementary figure S1).

**Table 5:** Validation data of closest flanking marker on Australian cereal cultivars

	**Cultivar**	***sunCS_YR63***
Controls	AUS27955 (*YR63)*	G:G
	AUS27928S	C:C
Hexaploid Wheat	Anapurna, Axe, B53, Beckom, Borlaug 100, Bremer, Buchanan, Calingiri, Catapult, Chara, Chief CL Plus, Condo, Coolah, Corack, Correll, Cosmick, Cutlass, Derrimut, Devil, DS Bennett, DS Darwin, DS Faraday, DS Pascal, DS Tull, EG Jet, EG Titanium, EGA Bounty, EGA Eagle Rock, EGA Gregory, EGA Kidman, EGA Wedgetail, Einstein, Elmore CL Plus, Emu Rock, Espada, Estoc, Forrest, Grenade CL Plus, Harper, Hartog, Hatchet CL Plus, Hydra, Illabo, Impress CL Plus, Jade, Janz, Justica CL Plus, Kinsei, Kiora, Kord CL Plus, LGGold, Livingston, Longsword, LRPB Arrow, LRPB Beaufort, LRPB Cobra, LRPB Dart, LRPB Flanker, LRPB Gauntlet, LRPB Gazelle, LRPB Havoc, LRPB Hellfire, LRPB Impala, LRPB Kittyhawk, LRPB Lancer, LRPB Mustang, LRPB Nighthawk, LRPB Nyala, LRPB Oryx, LRPB Parakeet, LRPB Reliant, LRPB Scout, LRPB Spitfire, LRPB Trojan, Mace, Magenta, Manning, Merlin, Mitch, Morocco, Naparoo, Ninja, Orion, Phantom, Preston, Razor CL Plus, RGT Accroc, RGT Calabro, RGT Ivory, RGT Zanzibar, RockStar, Scepter, SEA Condamine, SF Adagio, SF Hekto, SF Ovalo, SF Scenario, Shark, Sheriff CL Plus, Shield, SQP Revenue, Steel, Strzelecki, Sunchaser, Sunguard, Sunlamb, Sunmate, Sunmax, Sunprime, Suntime, Suntop, Sunvale, Supreme, Tenfour, Tungsten, Viking, Vixen, Wallup, Westonia, Wyalkatchem, Yitpi, Zen, Zircon (EDGE06-039-13)	C:C
Durum	Bitalli, Caparoi, DBA Artemis, DBA Aurora, DBA Bindaroi, DBA Lillaroi, DBA Spes, DBA Vittaroi, EGA Bellaroi, Hyperno, Jandaroi, Penne, Rotini, Tjilkuri, Westcourt	C:C
Triticale	Astute, Berkshire, Bison, Canobolas, Cartwheel, Chopper, Endeavour, Fusion, Goanna, Joey, Kokoda, Normandy, Wonambi, Yowie	C:C

## Discussion

The persistent threat posed by *Pst* has triggered a global and extensive endeavour aimed at identifying and characterizing valuable resistance (*R)* genes in wheat. To ensure the ongoing protection of wheat production in Australia, where *Pst* incursions have been a recurring issue, it is imperative to test both existing and novel *R* genes against local and international *Pst* pathotypes. Landraces, that have adapted to specific geographical regions over time, present a valuable resource for discovering novel genes for diverse breeding traits [18].

In this study, we investigated the efficacy of *YR63* against a range of Australian and International *Pst* pathotypes using the *YR63* donor landrace accession AUS27955. Previous studies indicated that *YR63* exhibited a strong resistance against Indian *Pst* pathotypes [19], alongside *Yr47* and *Yr57*, all of which have been identified in the Watkins Wheat Landrace Collection [20, 21]. Indian wheat production heavily relied on the resistance provided by *Yr9*, *Yr17* and *Yr27*, but the local *Pst* pathotypes such as 46S119, 110S119 and 238S119 evolved to acquire virulence against these genes [19, 22].

Our findings revealed that *YR63* confers resistance against global pathotypes of *Pst* representing Africa, Asia, Middle East and South America. Furthermore, we also observed that *YR63* provides resistance against *Pst*S11, detected in Afghanistan in 2012, and *Pst*S17, first observed in Egypt in 2018 [23, 24]. *Pst*S11 has spread to several countries in the Middle East and Africa; including Turkey, Ethiopia, and Kenya, while *Pst*S17 has been observed in Middle East, Turkey, Ethiopia and Baltic countries [25]. Critically, *YR63* can also defend against *Pst*S1 and *Pst*S2, two of the most important *Pst* lineages, globally. In eastern Australia, *Pst*S13 has been dominant in wheat production areas [2], but *YR63* demonstrated high level of resistance against this pathotype under field conditions. Considering the broad spectrum of resistance exhibited by *YR63* against currently prevalent global *Pst* pathotypes, this gene represents a valuable resource for international wheat breeding programs. The in-effectiveness of *YR63* against *Pst*S7, *Pst*S9, *Pst*S14 and 239 E237 A- 17+ 33+ (*Pst*S10) indicates the necessity for the continuous search of novel *ASR* genes through mining of highly diverse germplasm such as the Watkins Collection.

In this study, we also confirmed the short arm of Chr 7B as the chromosomal location of *YR63*. This chromosome is known to carry stripe rust *ASR* genes *Yr2*, *Yr6*, and *Yr67* and *APR* genes *Yr39*, *Yr52*, and *Yr59* [26]. Considering the virulence profiles of *Pst* pathotypes used, it was concluded that AUS27955 does not carry *Yr2*, *Yr6*, *Yr39*, *Yr52*, or *Yr59*. *YR63* differed from *Yr67* for its in-effectiveness against the *Pst* pathotype 239 E237 A- 17+ 33+ [27]. Further, in the in-depth genome analysis of the locus, a cluster of 16 *NLR* genes and 6 *kinase* genes was detected in the homologous region (0.9 to 4.0 Mb of chromosome 7B) of *YR63*. It is worth noting that gene clustering has been observed in various organisms, including prokaryotes and eukaryotes. Although operons are commonly associated with prokaryotes [28], gene clusters in plants are typically attributed to homologous gene duplications or functionally linked non-homologous genes [29, 30]. In the case of wheat, *NLR* clusters have been observed in resistance genes including the stem rust gene *Sr50* which contains an additional six homologous *NLRs* flanking the *R* gene [31].

Although the identification of gene clusters can assist in pinpointing favourable regions for gene selection, the presence of homologous elements hinders map-based cloning and inhibits sequencing techniques that offer comprehensive genomic insights. In the case of *Lr1*, cloning endeavours were impeded by the absence of specific markers tailored to the Chr 5D cluster [32]. Similarly, our efforts to narrow down this region using RNASeq and MutRenSeq failed to produce additional markers that reliably segregated with the *YR63* phenotype.

This study utilised short-read next-generation sequencing (SR-NGS) with read lengths up to 150 bp. SR-NGS presents benefits such as diminished error rates, increased data yield, and cost-effectiveness when contrasted with long-read sequencing techniques; nevertheless, the 10 kb capacity of long-read sequencing holds the potential to enhance the resolution of the *YR63* locus by mitigating the presence of multiple target sites within the *YR63* locus [33]. The SR-NGS sequencing also offer the faster identification of SNP-based markers linked with the target trait.

This study successfully identified the marker *sunCS_YR63* from the MutRenSeq dataset to effectively distinguish AUS27955 (*YR63*) from a comprehensive collection of 152 Australian bread and durum wheat and triticale cultivars. Availability of linked molecular markers is critical for pyramiding particularly for *R* genes with similar phenotype against diverse pathotypes of the target pathogen [34]. The *YR63*-linked marker *sunCS_YR63* can be used for marker assisted selection of this gene in wheat breeding programs. However, due to the intricate and highly repetitive nature of the *YR63* locus, long-read sequencing techniques as demonstrated in the cloning of *Yr27* [35] may be more suitable for unravelling their complexities and distinguishing the candidate gene for the *YR63* mediated resistance. This may also assist in future attempts to generate additional KASP markers within the *YR63* locus.

## Conclusion

In summary, our study demonstrates that the *YR63* gene exhibits robust resistance against a wide range of *Pst* pathotypes from diverse global regions, making it a valuable asset for international wheat breeding programs. We located the *YR63* gene on the short arm of chromosome 7B, alongside a cluster of *NLR* and *kinase* genes, which can aid in gene selection but present challenges for map-based cloning and sequencing. While our research employed short-read next-generation sequencing (SR-NGS) with its advantages in data quality and cost-efficiency, long-read sequencing techniques may offer a more comprehensive view of the complex and repetitive *YR63* locus. The identification of the *YR63*-linked marker *sunCS_YR63* provides a practical tool for marker-assisted selection in wheat breeding programs, especially when pyramiding resistance genes against diverse *Pst* pathotypes is required.

## Methods

### Plant materials

Landrace accession AUS27955, carrying *YR63*, is the resistant parent and positive control for all experiments, while AUS27928S, a selection from accession AUS27928 (AGG No: AGG27928WHEA1) lacking *YR63* or any earlier known *ASR* genes for stripe rust was used as the susceptible parent. The mapping population consisted of 195 RILs generated from an initial crossing of AUS27955 with AUS27928S, single plant progeny was progressed forward. A mutant population was generated from the resistant parent, AUS27955. A kill-curve consisting of 0%, 0.2%, 0.4%, 0.6% and 0.8% EMS solution was applied to a small set of seeds (10-15) and grown in a glasshouse. The seed treatment that generated an approximate 50% reduction in germination and height of treated wheat was used. For the mutant population. ~2000 seeds were mutagenized with the chosen EMS solution following the procedure described by Mago et al. (2017) [36].

### Rust inoculation and disease screening

Plant material was sown in 9 cm diameter plastic pots (12-16 plants per line), with a composite potting mixture of 80% composted pine bark and 20% sand. Aquasol^®^ was applied to material at a rate of 20 g per 10 L of water. Both parent lines and ‘Morocco’ were sown as control lines for plant inoculation. The Australian *Pst* pathotypes were screened using inoculum at the Plant Breeding Institute, University of Sydney. *Pst* pathotype 134 E16 A+ 17+ 27+ (*Pst*S1) which is avirulent on *YR63* was used for screening the mapping population for gene segregation and marker-trait linkage analysis*.* The *Pst* pathotypes, 198 E16 A+ J+ T+ 17+ (*Pst*S13) and 239 E237 A- 17+ 33+ (*Pst*S10) were also used to test the parental accessions. Plants were inoculated at the two-leaf stage by spraying with urediniospores suspended in light mineral oil (Isopar L, approx. 5 mg spores per 10 mL oil). The plants were incubated in plastic-covered steel trays filled with water (a dew chamber) for 24 h at 9 °C before being moved to a greenhouse maintained at 17 °C. Stripe rust disease severity was scored at 12-14 days post-inoculation using the ; to 4 scale described by McIntosh et al. (1995) [37]. Parallelly, to check the broad-spectrum effectiveness of *YR63*, the two parental accessions were also screened against global *Pst* isolates representing *Pst*S2*, Pst*S7*, Pst*S8*, Pst*S9*, Pst*S10*, Pst*S11*, Pst*S13*, Pst*S14 and *Pst*S17 at the Global Rust Reference Center (GRRC), Denmark (Table 1) using the procedures described in Hovmøller et al. (2017) [38]. A full list of the avirulence/virulence profiles of each tested *Pst* pathotype can be found in Supplementary table S1.

### DNA extraction and marker analysis

DNA was extracted from the RIL mapping population using a Hamilton Microlab^®^ NIMBUS automated liquid-handling robot and the procedure outlined in Kota et al. (2006) [39]. Approximately 2 cm of leaf tissue was collected from seedlings and ground in a Qiagen Tissue lyser II. The contents were then settled by centrifugation and DNA extraction buffer was added. The plates were incubated at 65 °C, cooled, and 6M ammonium acetate was added. The plates were centrifuged, and the supernatant was recovered into new deep-well microtiter plates containing isopropanol. The DNA was allowed to precipitate, then the plates were spun and washed in 70% ethanol. The pellets were allowed to fully dry before being resuspended in distilled water. The plates were centrifuged, and the supernatant was transferred to new microtiter plates for use in experiments.

### Mapping through marker-trait linkage analysis

Genomic DNA from a subset of 115 RILs selected randomly from AUS27955 x AUS27928S cross were sent to Centre for AgriBioscience, Victoria, Australia for tGBS analysis. SNPs from the tGBS scaffold markers associated with *YR63* resistance were converted into KASP markers to genotype the 195 individual lines of the RIL population*.* The automated pipeline Polymarker was used to assist in designing specific KASP markers identified. Markers were first screened on AUS27955 and AUS27928S before screening on the entire mapping population using the protocol described in Nsabiyera et al. (2016) [40]. Marker fluorescence was measured using a CFX96 Touch real-time PCR machine (Bio-Rad Laboratories Pty. Ltd., USA).

A Chi-squared (χ^2^) test was performed to confirm the inheritance of genes in the mapping population. Genetic distance was calculated using the Kosambi formula [41] available in the ‘onemap’ package [42] on RStudio 2022.02.2 [43] and was constructed using MapChart v2.32 [44].

### RNASeq analysis

Three days after inoculation, leaf samples were collected from AUS27955 and AUS27928S and was immediately frozen in liquid nitrogen and stored at -80 °C for later use. Whole RNA was extracted using the Maxwell^®^ RSC Plant RNA Kit (Promega) using the manufacturers protocol on the Maxwell^®^ RSC instrument. RNA samples were sent to Novogene for paired end read sequencing. Quality of the raw RNA reads was assessed using the FastQC and trimmed using the Trimmomatic tool to remove highly repetitive sequences, adapter sequences or redundant sequences. A de novo assembly of the RNA sequences was performed using CLC Genomics software (v21), which produced a new set of transcripts representing the expressed genes in the leaf tissue. The trimmed RNA reads from both the resistant and susceptible plant lines were then aligned to this assembled transcriptome using a tool called Burrows-Wheeler Aligner v0.7.17 [45]. The module, SAMtools (v1.12) [46], was used to generate reads counts for individual transcripts, following which the read counts were normalised and calculated to reads per million.

### MutRenSeq analysis

High quality DNA from the resistant accession AUS27955 and four loss-of-function mutants was sent to Arbor Biosciences (https://arborbiosci.com/) for enrichment and sequencing of DNA fragments related to *NLR*s. Targeted gene enrichment was based on the MYbaits protocol and bait library described in github.com/steuernb/MutantHunter. The sequence capture data supplied by Arbor Biosciences was processed as per the pipeline described by Steuernagelet al. (2016) [6]. First, the raw data was analysed for quality using FastQC (https://www.bioinformatics.babraham.ac.uk/projects/fastqc/). The reads were trimmed of adapters, repeat sequences, and low-quality regions using Trimmomatic [47] based upon the FastQC output. The wild-type sequences were assembled using the de-novo assembly tool in the CLC Genomics Workbench (https://digitalinsights.qiagen.com) using a minimum fragment length of 300, length fraction of 0.95, and a similarity fraction of 0.98. This served as a reference genome for the mutants. The trimmed reads (from both the mutants and wild-type) were mapped to the wild-type assembly using the Burrows-Wheeler Aligner [45]. Background noise was removed from each alignment using the program Noisefinder.pyc, then SNPs were called using SNPlogger.pyc. Another program, SNPtracker.pyc, was then used to generate a report summarising which contigs were polymorphic. Candidate contigs were shortlisted based on the presence of mutations in the maximum number of mutant lines screened. These custom programs (Noisefinder.pyc, SNPlogger.pyc and SNPtracker.pyc) were developed in-house, and are available on GitHub (https://github.com/TC-Hewitt/MuTrigo).

Using the wild-type assembly as a reference, SNPs were identified between AUS27955 and AUS27928S, then converted to KASP markers. The markers were used to screen the RIL population to determine whether candidates co-segregated with the *YR63* phenotype.

### Comparative genomic analysis of *YR63* locus

To understand genomic architecture of *YR63* locus, marker positions were first identified in Chinese Spring genome assembly v2.1. Matching positions were identified in publicly available genomes of Arina*LrFor*, CDC Landmark, CDC Stanley, Jagger, Julius, LongReach Lancer, Mace, Norin 61, SY Mattis, PI190962 (spelt wheat), Zavitan and Svevo [11, 13]. The module BLAST+ (2.12.0) [48] was used to compare genes and sequences to identify homologous genes.

The *NLR* and kinase gene sequences from the *YR63* locus were separately aligned using the Clustal-Omega platform using default settings (https://www.ebi.ac.uk/Tools/msa/clustalo/). The separate phylogenetic trees were constructed using the software FigTree (v1.4.4, https://github.com/rambaut/figtree/releases).

### Supplementary Information

**Additional file 1: Supplementary table S1.** Virulence/avirulence profiles of *Pst *pathotypes.** Supplementary figure S1.** Marker segregation of *sunCS_YR63 *on Australian Cereal cultivars. Blue indicates AUS27955. Orange indicates susceptible/other allele from the Australian cereal cultivars.

## Data Availability

The datasets of raw Illumina sequences generated in the current study were deposited to the National Center for Biotechnology Information (NCBI) and can be accessed in the Short Read Archive (SRA) database (https://www.ncbi.nlm.nih.gov/sra) as accession number PRJNA988831. Seeds of plant materials used in this study are available from the corresponding author by request.
