# Supplementary material for: Wheat stripe rust resistance locus YR63 is a hot spot for evolution of defence genes – a pangenome discovery
Source: BMC Plant Biol. 2023 Nov 27;23:590. doi: 10.1186/s12870-023-04576-2 (PMC10680240; doi:10.1186/s12870-023-04576-2)
Supplement: Supplementary file 1 — Additional file 1: Supplementary table S1. Virulence/avirulence profiles of Pst pathotypes. Supplementary figure S1. Marker segregation of sunCS_YR63 on Australian Cereal cultivars. Blue indicates AUS27955. Orange indicates susceptible/other allele from the Australian cereal cultivars. [file 12870_2023_4576_MOESM1_ESM.docx]

**Supplementary table S1.** Virulence/avirulence profiles of *Pst* pathotypes.

| **Region** | **Genetic Group** | **Avirulence** | **Virulence** |
| --- | --- | --- | --- |
| Australia | *Pst*S1^a^ | 1,3,4,5,10,15,24,32,Sp,Amb | 2,6,7,8,9,17,25,27,AvS |
|  | *Pst*S10^b^ | 5,8,10,15,24,27,Amb | 1,2,3,4,6,7,9,17,25,32,33,Sp,AvS |
|  | *Pst*S13^c^ | 1,3,5,10,15,24,25,27,Sp,Amb | 2,4,6,7,8,9,17,32,AvS,J,T |
| Global | *Pst*S2 | 3,4,5,10,15,17,24,32,Sp,Amb | 1,2,6,7,8,9,25,27,AvS |
|  | *Pst*S7 | 5,8,10,15,24,27 | 1,2,3,4,6,7,9,17,25,32,Sp,AvS,Amb |
|  | *Pst*S8 | 4,5,10,15,24,Sp | 1,2,3,6,7,8,9,17,25,32,AvS,Amb |
|  | *Pst*S9 | 5,7,8,10,15,17,24,Sp,AvS,Amb | 1,2,3,4,6,9,25,27,32 |
|  | *Pst*S10 | 5,8,10,15,24,27,Amb | 1,2,3,4,6,7,9,17,25,32,Sp,AvS |
|  | *Pst*S11 | 1,3,5,9,10,15,24,25,Sp,Amb | 2,(4),6,7,8,17,27,32,AvS |

Australian pathotype classification: ^a^134 E16 A+ 17+ 27+, ^b^239 E237 A- 17+ 33+, ^c^198 E16 A+ J+ T+ 17+.


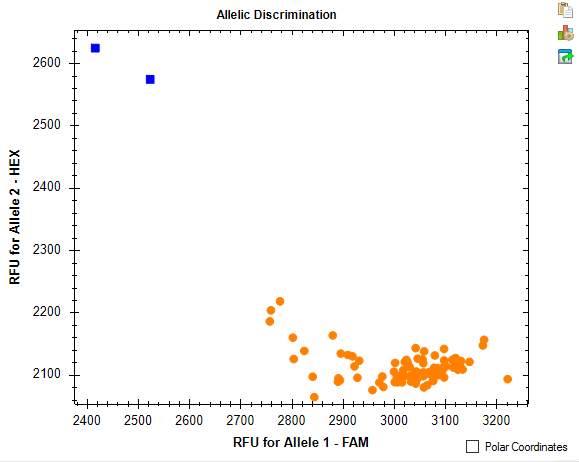


**A.**


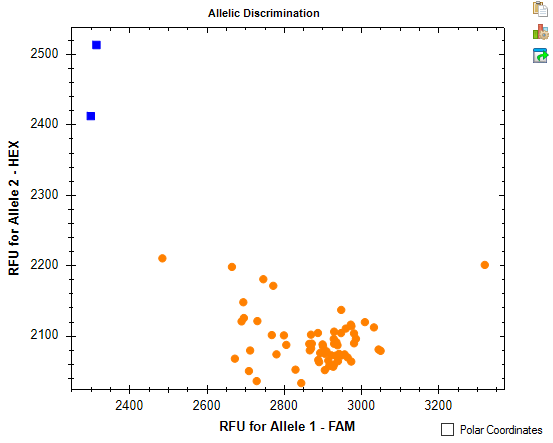


**B.**

**Supplementary figure S1.** Marker segregation of *sunCS_Yr63* on Australian Cereal cultivars. Blue indicates AUS27955. Orange indicates susceptible/other allele from the Australian cereal cultivars.
